# Accurate and Precise Coulometric Determination of Sulfur Dioxide in Compressed Gas Mixtures

**DOI:** 10.6028/jres.096.030

**Published:** 1991

**Authors:** G. D. Mitchell, A. A. Bell

**Affiliations:** National Institute of Standards and Technology, Gaithersburg, MD 20899

**Keywords:** acid rain, analysis of gases, compressed gas, coulometry, sulfur dioxide

## Abstract

Coulometry has been established as an important and reliable method for the determination of acidic compounds. The analytical method and simple apparatus described here arc applied to the precise and accurate determination of sulfur dioxide in nitrogen, specifically in compressed gas cylinders at nominal concentrations of 50 and 100 µmol/mol (ppm). This method is constant current coulometry where the magnitude of the current is set by the balance between the electrochemical generation of OH−, the flow of SO_2_, and the chemical reaction of the solution. The method is direct, rapid, and can be refined further to provide analysis at the nanomol/mol level.

## 1. Introduction

Sulfur dioxide (SO_2_) in the atmosphere is a common pollutant and is a major contributor to the formation of acid rain. Accurate and precise determinations of SO_2_ in the atmosphere are essential to determine the magnitude of the problem. Reference gas mixtures such as NIST SRMs are an important part of the measurement procedure. The method presented here is intended for the analysis of cylinder gas mixtures of SO_2_ in nitrogen. The determination of SO_2_ has been accomplished by a variety of techniques [1,2,3,4,5,6,7]. Most of the methods are time consuming, employ expensive instrumentation, and need sample pretreatment. The coulometric titration method described here, for the determination of SO_2_ in compressed gas mixtures of SO_2_/N_2_, is sensitive, requires a modest investment in instrumentation, is applicable to other reactive gases, and under explicit experimental conditions is independent of calibration standards.

The accuracy of constant current coulometry is dependent upon several factors: the Faraday, electrode processes, electrode kinetic parameters, and the current efficiencies. These critical factors and their importance in coulometry are discussed in detail in Ref. [7]. The coulometric determination of strong acids or bases most nearly approaches the ideal measurement situation [8,9,10] in aqueous media completely free of reactive impurities. However, no matter what the working conditions are, at least one secondary reaction occurs in competition with the desired electrode reaction, even though it may be very slight. The current efficiency is thereby, in the limit, set by the relative rate of the desired electrode reaction and the competing background electrode processes. The goal in constant current coulometry is therefore to characterize the current efficiency under specific experimental conditions, thus delineating any systematic bias associated with the method.

Calibration mixtures of SO_2_ in N_2_ in compressed gas cylinders are generally characterized by using precise instrumental techniques that have been calibrated with accurately prepared standards, or with standards that have been characterized using independent reference methods. The reference method for SO_2_ analysis is acid-base titration accomplished by the absorption of SO_2_ gas by a solution of hydrogen peroxide (H_2_O_2_) followed by the titration of the resultant H_2_SO_4_ with a standard base [11]. The method, however, has three inherent sources of error: 1) the measurement of the volume of gas mixture passed through the absorping solution, 2) the accuracy associated with the standard base, and 3) the imprecision of titration. To overcome these limitations, a coulometric based titration system with optical detection has been developed.

The coulometric method described here provides experimental data that are directly related to the Faraday constant. To evaluate the accuracy of the method, it is necessary to evaluate the various aspects of the overall approach. The three criteria that define the approach are:

- a quantitative reaction scheme for SO_2_
- accurate SO_2_ sample delivery
- an independent determination of the current efficiency.

The reaction scheme selected for the coulometric determination of SO_2_/N_2_ is analogous to that used by the Peroxide Method [11]. The coulometry-based acid-base neutralization is accompanied by both complete absorption of the analyte and near 100% current efficiency.

## 2. Experimental Methods

### 2.1 Reagents

All solutions are prepared from reagent grade materials and distilled H_2_O that has been passed through an adsorption/ion exchange filtration process. The absorption solution is prepared by dissolving 20 g of KCl in water and adding 5 mL of 30% hydrogen peroxide and 25 mL of a bromcresol green solution (0.1% by volume), followed by dilution to 1 L. The pH of the solution is adjusted to 4.0 by the addition of 1% hydrochloric acid, if needed. A 2% KCl solution adjusted to a pH of 4.0 was prepared for the anode compartment of the electrochemical cell. The cell compartments are separated by a sintered glass disc and a 3% KCl-Agar plug.

### 2.2 Apparatus

**Figure 1:**
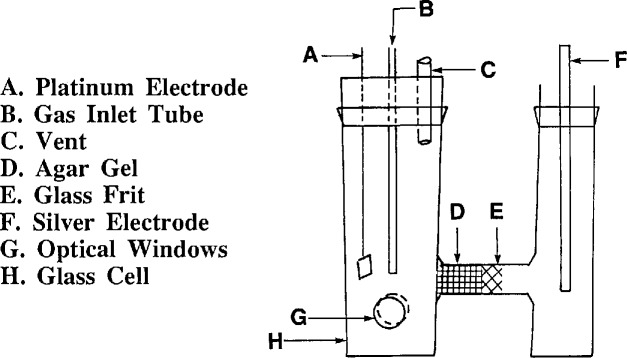
Coulometrie titration cell.

The coulometric system consists of essentially three components: a reaction chamber (the cell), an absorption solution, and an amplified photometric feedback circuit for end-point detection. The reaction chamber is an “H-base” glass cell with two electrode compartments separated by a glass frit and a salt-agar bridge (Fig. 1). The hydrogen peroxide absorption solution contains a pH indicator and the electrolyte. Detection of the coulometric titration end-point is achieved through a current feedback circuit. The feedback current to the working electrode is supplied by the optical detection system which consists of photodiodes and interference filters. The method calls for a silver rod anode (7 mm diameter, 100 mm length) and a platinum cathode with 2 cm^2^ surface area. The cathode compartment is 31 mm in diameter and 95 mm in height. The compartment contains two windows 15 mm in diameter for use with the optical detection system. The compartment is fitted with a rubber stopper through which the platinum electrode and a gas delivery tube are inserted. The anode compartment, 95 mm in height and 25 mm in diameter, contains the silver rod electrode and the 2% KCl electrolyte solution. The cathode and anode compartments are filled approximately 50 mm above their bases with the absorption solution and the 2% KCl electrolyte, respectively.

The dc amplifier circuit [12] operates by using the output signal from the photodiode detectors as a titration current through a feedback loop. The system is powered by a 15 V regulated power supply. An auto-ranging DVM with an IEEE interface is used to monitor the current output from the titration cell.

A mass flow controller (MFC) capable of measuring gas flow rates up to 100.0 ± 0.1 mL/min is used. The MFC is periodically calibrated using a wet test meter that has been calibrated by gravimetry.

A three-way solenoid valve is used to direct the flow of sample gas or zero gas (N_2_) through the reaction cell.

A 10 turn potentiometer is used to control the nulling circuit, which balances the detectors for ambient light conditions. An on/off switch empowers the detector light source.

The optical system consists of two 620 nm interference filters, two photodiodes, a single biconvex glass lens, and a dc lamp, all mounted on an optical bench.

The entire system is controlled by a computer.

### 2.3 Procedure

**Figure 2:**
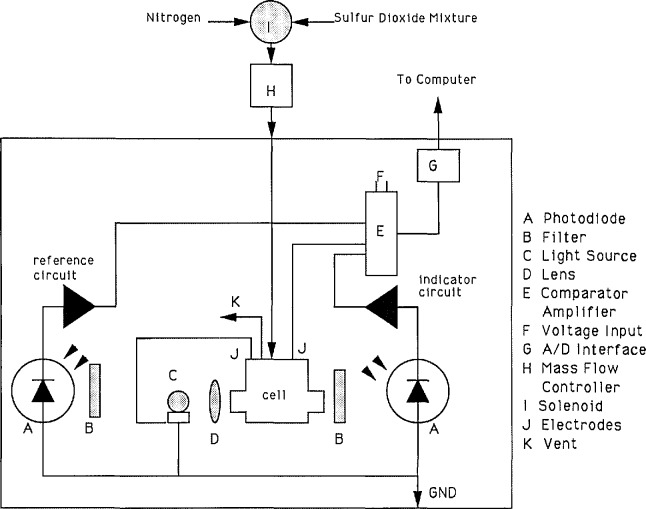
Diagram for apparatus.

The absorption cell compartments are filled with the appropriate solutions. The cell is placed in the optical system (Fig. 2) so that the light from the lamp strikes the cell at the window and passes through the absorption solution without any obstructions. The appropriate connections are made to the gas inlet tube, and to the anode and cathode of the cell. The cover is placed over the cell to eliminate as much background light as possible. The current flow is adjusted to zero using the nulling potentiometer and SO_2_-free zero gas (N_2_) is allowed to flow through the cell. At this point the system is ready, and the entire analysis can be controlled by the computer.

The computer is interfaced to a current meter in series with the cell and a printer. The computer control is a menu driven central utility program that not only controls the experiment, but also has data reduction capabilities. The Central Control program is selected, all necessary parameters are specified (date, cylinder identification, barometric pressure, temperature, type of analysis (SO_2_), estimated concentration, flow rate of gas, number of replicates, and the disk storage file) and the program is started. The computer energizes a solenoid, thus selecting the analyte gas mixture. During the analysis, time (min) and current (mA) are stored in the run file at 30 s intervals for 15 min. At the end of 15 min, the program collects 200 current readings of the cell in a 2 min interval and reports the mean value as the cell current. The solenoid is de-energized and nitrogen gas is passed through the cell, decreasing the cell current to a predetermined level, where the software either replicates or ends the analysis.

The method described for the determination of SO_2_(g) in a N_2_ matrix is based on the following series of reactions:

$${\text{SO}}_{2}+H_{2}O_{2}=2H^{+}+{{\text{SO}}_{4}}^{=}\;\;(\mathrm{solution})$$ (1)

$$2H_{2}O+2e^{-}=2{\text{OH}}^{-}+H_{2}\;\left(\mathrm{cathode}\right)$$ (2)

$${\text{OH}}^{-}+H^{+}=H_{2}O\;\left(\mathrm{solution}\right)$$ (3)

$$\mathrm{Ag}+{\mathrm{Cl}}^{-}=\mathrm{AgCl}+e^{-}\;\left(\mathrm{anode}\right)$$ (4)

Sulfur dioxide reacts with the hydrogen peroxide to form sulfuric acid. The acid causes a change in the color of the indicator in the absorption solution, thus bringing about a change in the electronic null circuit. The shift in pH and color of the absorption solution causes an increase in the amount of light striking a photodiode detector whose output is fed back to the cell as a titration current. At the cathode, hydroxide is produced to neutralize the acid formed in reaction [1]. The rate of formation of hydroxide at the cathode is equivalent to the rate of formation of the sulfuric acid. At steady state the current is proportional to the original SO_2_ concentration of the sample gas. The accuracy of the entire process is dependent upon two factors 1) stoichiometric conversion of SO_2_ to H_2_SO_4_ and 2) near 100% current efficiency.

## 3. Results and Discussion

The absorption efficiency of SO_2_ in 1.5% H_2_O_2_ was determined during “wet chemistry” titration using the reference method [11]. Using dual impingers arranged in series, 15 L of 100 ppm SO_2_/N_2_, at a flow rate of 1 L/min, were passed through the two impingers, each containing 20 mL of 1.5% H_2_O_2_. Analysis of the contents of the first impinger showed that the amount of SO_2_ in the flowing stream was 100 ppm. A titration of the contents of the second impinger revealed no H_2_SO_4_, which means that all of the SO_2_ was absorbed in the first impinger. In this system the sample gas flows at a rate of 50 mL/min for about 15 min, which amounts to less than 1 L of SO_2_/N_2_ mixture per determination. The working electrode compartment of the cell (Fig. 1) is similar to the impingers used for the absorption studies.

Current efficiency studies were performed using a permeation tube calibration system consisting of a thermostatically-controlled chamber containing a gravimetrically-calibrated SO_2_ permeation device and a calibrated mass flow controller. The calibrated permeation tube serves as an an accurate and independent source of SO_2_ gas. From the known output of the permeation tube and calibrated flow of dry N_2_ or air over it, concentrations of SO_2_ are obtained with an uncertainty of 0.5%. The results of the current efficiency study are summarized in Table 1. Many factors contribute to inaccuracies in the generation and measurement of standards. These include: 1) a bias in the current measurement, 2) a bias in the critical components of the electronic circuit, 3) the interference of a competing background reaction, and 4) the imprecision of the dynamic dilution system. Based on the result presented in Table 1, the current efficiency is considered to be 101.1 ± 0.6%, within the limit of its experimental determination. As mentioned before, the coulometric determinations of strong acids or bases most nearly approach the ideal measurement situation [8,9,10] in aqueous media completely free of reactive impurities. A current efficiency of 100% is only obtainable for one type of electrode reaction: the electrolysis of a molten salt or of a pure solvent; and this only holds when the products of the anode and cathode reactions are prevented from reaching the other electrode. Therefore, except for the ideal case, there is always at least one secondary competing reaction at the electrode [13]. The estimated uncertainty of the SO_2_ concentration from the dynamic dilution system is approximately ±0.5%. For the coulometric method the 101.1% current efficient is a measure of a systematic bias in the analysis for which a correction has to be applied.

Results of a series of analyses of SO_2_/N_2_ Standard Reference Materials (SRM), at the nominal 50 and 100 ppm level are given in Table 2. The certified values are concentrations placed on the individual SO_2_/N_2_ mixtures as determined by acid-base peroxide titration [11] and non-dispersive infrared analysis. They are presented here only as a frame of reference for the coulometric values. The uncertainties reported for the coulometrically determined cylinders in Table 2 are representative of the overall analytical imprecision at the 95% level of confidence. These uncertainties are the result of the uncertainty placed on the method at its present stage of developement following the long-term study of the control cylinder (cylinder A, Table 2). The uncertainty of a single analysis of cylinder gas mixtures of SO_2_ in N_2_ at the 50 to 100 µmol/mol level is ± 6% at the 95% confidence level.

To evaluate this particular method for its independence, and as a companion to the standard reference method [11], one must look critically at the factors that are important. The equation used in the final calculation of concentration is:

$${\mathrm{SO}}_{2}\left(\frac{{\mu \mathrm{mol}\;\mathrm{SO}}_{2}}{{\mathrm{mol}\;N}_{2}}\right)=\left(\frac{C_{\mathrm{cell}}\cdot K}{Q}\right)$$ (1)

where

*C*_cell_ = Cell current (A)
*Q* = Flow rate of sample (mL/min)

and

$$K=\frac{6\cdot 10^{7}\cdot MV_{T}}{n\cdot F}$$ (2)

*MV_T_* = Molar volume of air (corrected for temperature) mL/mol (24470.69 @ 298 K)
*n* = Number of electron change (e^−^) (*n* = 2)
*F* =The Faraday constant (96485.38 A s/mol)
6·10^7^ is a unit conversion factor

$$\left(\frac{s\cdot \mu \mathrm{mol}}{\min\cdot \mathrm{mol}}\right)$$

From a review of Eq. (1), it becomes clear that the two important experimental variables are current and flow rate. To assure the accuracy of these two variables the current meter and mass flow controller described earlier are periodically calibrated. The coulometric method is capable of both precision and accuracy. This is supported by the data presented in Table 2. Once the bias correction is applied to the coulometric results the difference between the coulometric values and the certified values for all of the samples fall within the 2% uncertainty placed on the cylinders by peroxide titration and NDIR. The absolute difference between the corrected coulometric value and the certified value for all samples measured range from 0.2 to 0.4 µmol/mol.

## 4. Conclusion

The coulometric method described can be used to determine low concentrations (µmol/mol) of SO_2_ in nitrogen, at low flow rates and with a coulometric current efficiency near 100%. It offers a simple yet independent method for determining SO_2_/N_2_ in compressed gas cylinders at the µmol/mol level. The intercomparison of the results obtained by coulometry with those of the acid-base peroxide titration method shows that there is no statistical difference between the methods. However, coulometry is capable of the same degree of precision and accuracy as the acid-base peroxide titration method in less time and with less effort and, at the same time, gives results that are independent of calibration standards. The method presented here for the analysis of SO_2_/N_2_ compressed gas cylinders is easily adaptable to other analyte species such as CO, CO_2,_ NH_3_, NO_2_, H_2_S, and others. In the companion paper the method is modified for the analysis of cylinder gas mixtures of CO_2_ in Air.

## Figures and Tables

**Table 1:** Current efficiency determination^a^

Permeating tube concentration (µmol/mol)	Coulometric concentration (µmol/mol)	Current efficiency (%)
48.0 ± 0.6	48.6 ± 0.2	101.1 ± 0.6

a $\text{Current}\;\text{efficiency}=\frac{\text{Coulometric}\;\text{current}\;\left(\text{mA}\right)}{\text{Theoretical}\;\text{current}\;\left(\text{mA}\right)}\times 100$.

All uncertainties are expressed at the 95% confidence level.

**Table 2:** Coulometric titration results

Cylinder identification	Coulometric^a^ value (µmol/mol	Coulometric^c^ value (µmol/mol)	Certified^b^ value (µmol/mol)	Ratio (corrected to certified)
A	47.5	47.0	46.7	1.006
B	48.0	47.5	46.7	1.017
C	49.5	49.0	48.2	1.017
D	97.2	96.1	95.7	1.004
E	96.4	95.3	95.7	0.996

a The uncertainty at the 95% confidence level is 6%.

b The uncertainty at the 95% confidence level is 2%.

c Corrected for current efficiency bias.
